# The complete chloroplast genome sequence and phylogenetic analysis of *Ranunculus kadzusensis* Makino 1929, an endangered species in Korea

**DOI:** 10.1080/23802359.2025.2519219

**Published:** 2025-06-17

**Authors:** I-Jin Choi, Hey-Min Park, Wan-Hee Lee, Mi-Sung Park

**Affiliations:** Plant Management & Research Division, Seoul Botanic Park, Seoul, Republic of Korea

**Keywords:** Ranunculaceae, *Ranunculus kadzusensis*, chloroplast genome, plastome, phylogenetic relationships

## Abstract

*Ranunculus kadzusensis* Makino 1929 is one of the endangered endemic species on the Korean Peninsula. The complete chloroplast of *R. kadzusensis* is 158,301 bp in length with a typical quadripartite structure comprised of a large single-copy region (84,973 bp), a small single-copy region (17,638 bp), and two inverted repeat regions, each 27,845 bp in length. The overall GC content is 37.83%, the genome encoded a total of 113 genes, comprising 79 protein-encoding genes, 30 tRNA, and four rRNA genes. Phylogenetic analysis indicated that *R. kadzusensis* was closely related to *Ranunculus pekinensis* and *Ranunculus bungei*. This chloroplast genome enriches Ranunculus’s genome information and will also be valid for the species’ genetic classification in the family Ranunculaceae.

## Introduction

*Ranunculus* Linaeus 1753 is the largest genus in the family Ranunculaceae, with about 600 species widely distributed worldwide (Horandl et al. 2005). *Ranunculus* is recognized as monophyletic, comprising two subgenera and 17 series (Tamura 1993). *Ranunculus kadzusensis* Makino 1929 is an aquatic plant that grows as an annual or biennial grass. It is native to the West Coast and Gyeongju in Korea and distributed in Japan (Kim and Park 2022). *R. kadzusensis* is a common plant collected in Seoul, South Korea, until the 1960s. However, due to decreased arable land and urban expansion, the plant disappeared from its native habitat in the 1980s. It has been designated as an endangered plant in Korea since 2017 and has been protected. Recently, colonies in various parts of the country have been restored and protected. It is necessary to secure genetic information that can be distinguished from other Asian countries’ species. Section Batrachium within the genus *Ranunculus* consists of species primarily adapted to aquatic environments, commonly found in shallow freshwater habitats such as streams, ponds, and marshes. Species in this section typically exhibit finely divided, thread-like submerged leaves and floating leaves, with considerable morphological variation depending on environmental conditions. *R. kadzusensis* belongs to the section Batrachium along with *Ranunculus pekinensis* and *Ranunculus bungei*, sharing similar morphological and ecological characteristics, which indicate a close evolutionary relationship within this group (Wang and Tamura 2001). In recent years, the chloroplast genomes of some Ranunculus species have been published, such as *Ranunculus austro-oreganus* (Zeng et al. 2021), *Ranunculus cassubicifolius* (Karbstein et al. 2022), *Ranunculus japonicus* (Zeng et al. 2021), *Ranunculus macranthus* (Raubeson et al. 2007), *Ranunculus membranaceus* (Ren et al. 2023), *Ranunculus occidentalis* (Zeng et al. 2021), *R. pekinensis* (Liu et al. 2022), *Ranunculus sceleratus* (Kim et al. 2023), *Ranunculus ternatus* (Qiao et al. 2023), and *Ranunculus yunnanensis* (Rao et al. 2022). *R. kadzusensis* is an endangered species in Korea and Japan, yet its genetic and evolutionary relationships remain poorly understood. Chloroplast genome analysis is crucial in elucidating phylogenetic relationships, genetic diversity, and evolutionary patterns, essential for conservation biology and taxonomy. Despite the increasing availability of chloroplast genome data for *Ranunculus* species, no such data exist for *R. kadzusensis*, making this study a critical step toward filling this gap. Moreover, understanding the genetic characteristics of *R. kadzusensis* may contribute to conservation efforts, habitat restoration, and comparative genomic studies within the genus. This study aims to assemble and analyze the complete chloroplast genome of *R. kadzusensis* to determine its phylogenetic position. Furthermore, we discuss the broader implications of this research, particularly its significance for species identification and conservation strategies.

## Materials and methods

### Plant material, DNA extraction, and sequencing

**Figure 1.**
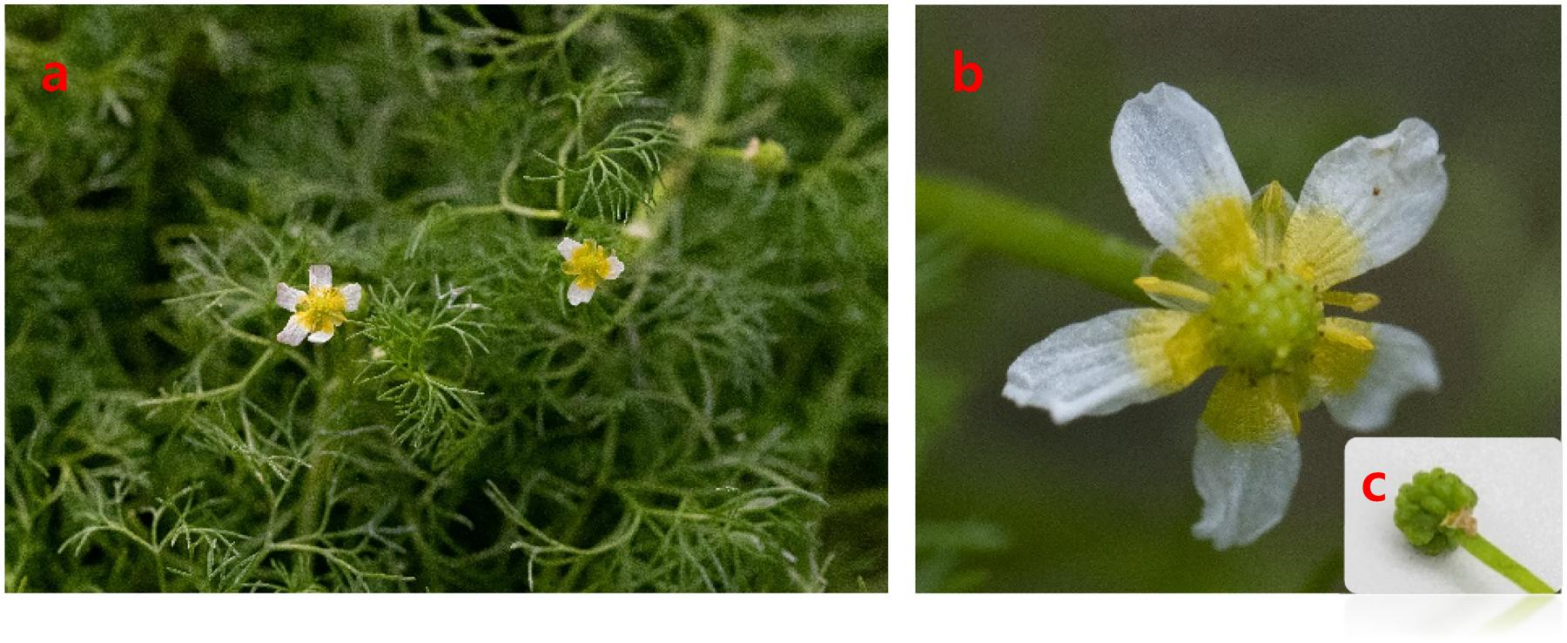
Species reference image of *R. kadzusensis*. This picture was taken by Hye-Min Park from Seoul Botanic Park, Seoul City, Korea (voucher number: 202300060; 37°34’12.9"N, 126° 50’08.1" E). **Core features**: The leaf is a thinly split thread-like submerged form (a), distinguishing it from *R. pekinensis* and *R. bungei* (Mu et al. 2024), which have wide floating and submerged leaves. The flowers are white (b), and the fruit is shaped like an inverted egg (c).

The species *R. kadzusensis* Makino specimen was sampled from Ongjin-gun, Incheon, Korea (37° 15′ 04.9″N, 126° 27′ 56.5″ E) and collected plant species that were cultivated and preserved at the Singu College Botanic Garden. A specimen was kept at Seoul Botanic Park (Hye-Min Park, plannerhm3@seoul.go.kr, number: 202300060) (Figure 1). We extracted DNA from 0.2 g of fresh leaf material using the i-genomic Plant DNA Extraction Kit (iNtRon, South Korea) and sequenced it on the Illumina NovaSeq 6000 platform. The sequencing produced 1.9 Gbps of raw paired-end data, which resulted in 1.6 Gbps of clean data after quality filtering to remove low-quality reads and adapter sequences. The filtered clean data was then assembled *de novo* using the dnaLCW method (Kim et al. 2015).

### Genome assembly and annotation

The trimmed data was assembled into contigs by using the CLC Assembly Tool (ver. 4.2.1, CLC Inc, Aarhus, Denmark). Using MUMmer (Kurtz et al. 2004), we identified contigs similar to the reference *Ranunculus* chloroplast genome and assembled them into a complete chloroplast genome. We annotated the genome with GeSeq (Tillich et al. 2017) and Artemis (Rutherford et al. 2000), followed by manual curation. Finally, we generated a chloroplast genome map with PMGmap (Zhang et al. 2024) and submitted the annotated genome to GenBank under the accession number PQ246022.

### Phylogenetic analysis

We analyzed the phylogenetic position of *R. kadzusensis* using chloroplast genome data from 19 Ranunculaceae species available in NCBI GenBank, using *Sinopodophyllum hexandrum* and *Berberis oiwakensis* (Berberidaceae) as outgroups. We extracted 67 shared chloroplast genes with Feature Extract (Wernersson 2005) and concatenated them into a single matrix (Table S3). A maximum-likelihood (ML) tree was constructed using MEGA 11 with 1000 bootstrap replicates, applying the Kimura 2-parameter model of nucleotide substitution (Tamura et al. 2021).

## Results

### General features of the chloroplast genome and IR boundaries analysis

**Figure 2.**
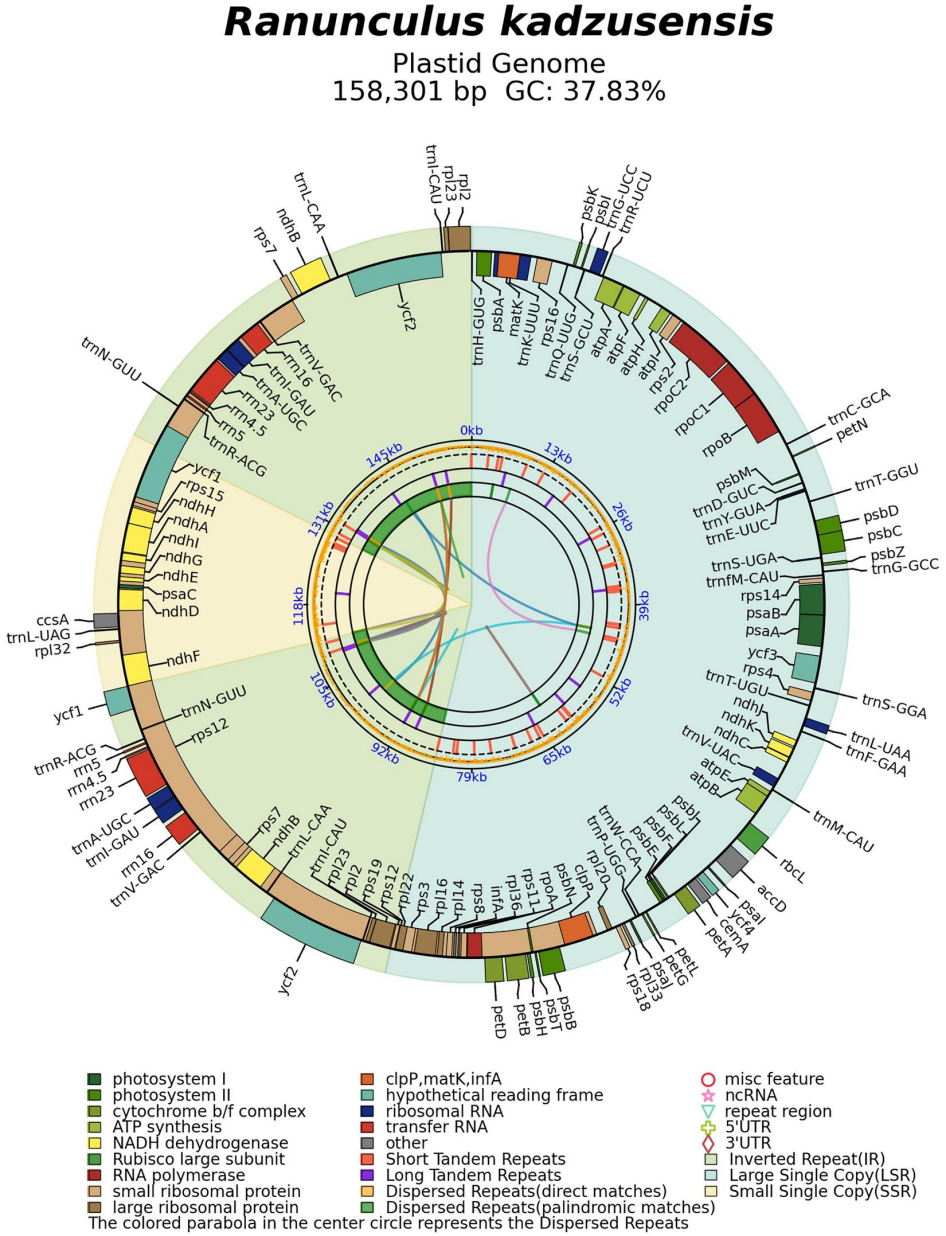
A circular map of the chloroplast genome, *R. kadzusensis*, was generated by PMGmap. The forward coding gene is outside the circle, and the reverse coding gene is inside the circle. The colored parabola in the center of the circle represents the dispersed repeats. The functional classification of the genes is provided in the bottom left corner of the figure (Tables S1, S2).

The chloroplast genome of *R. kadzusensis*(158,301 bp) exhibits a typical circular quadripartite structure, including an LSC region (84,973 bp), an SSC region (17,638 bp), and a pair of IRs (27,845 bp each) (Figure 2, Table S1). The coverage depth of *R. kadzusensis* is shown in Figure S1. Its GC content is 37.83%, with the IR region (42.55%) showing higher GC content than the LSC (36.11%) and SSC (31.25%) regions (Tables S1, S2). The sequencing depth of *R. kadzusensis* was analyzed to assess the genome assembly quality. The overall average sequencing depth was 183.08 ×, the maximum depth was 300 ×, and the minimum depth was 25× (Figure S1). Thirteen protein-coding genes are cis-splicing genes, with eleven containing one intron and two genes (*ycf3* and *clpP*) containing two introns. The *rps12* gene, on the other hand, has been recognized as a trans splicing gene (Figures S2, S3).

**Figure 3.**
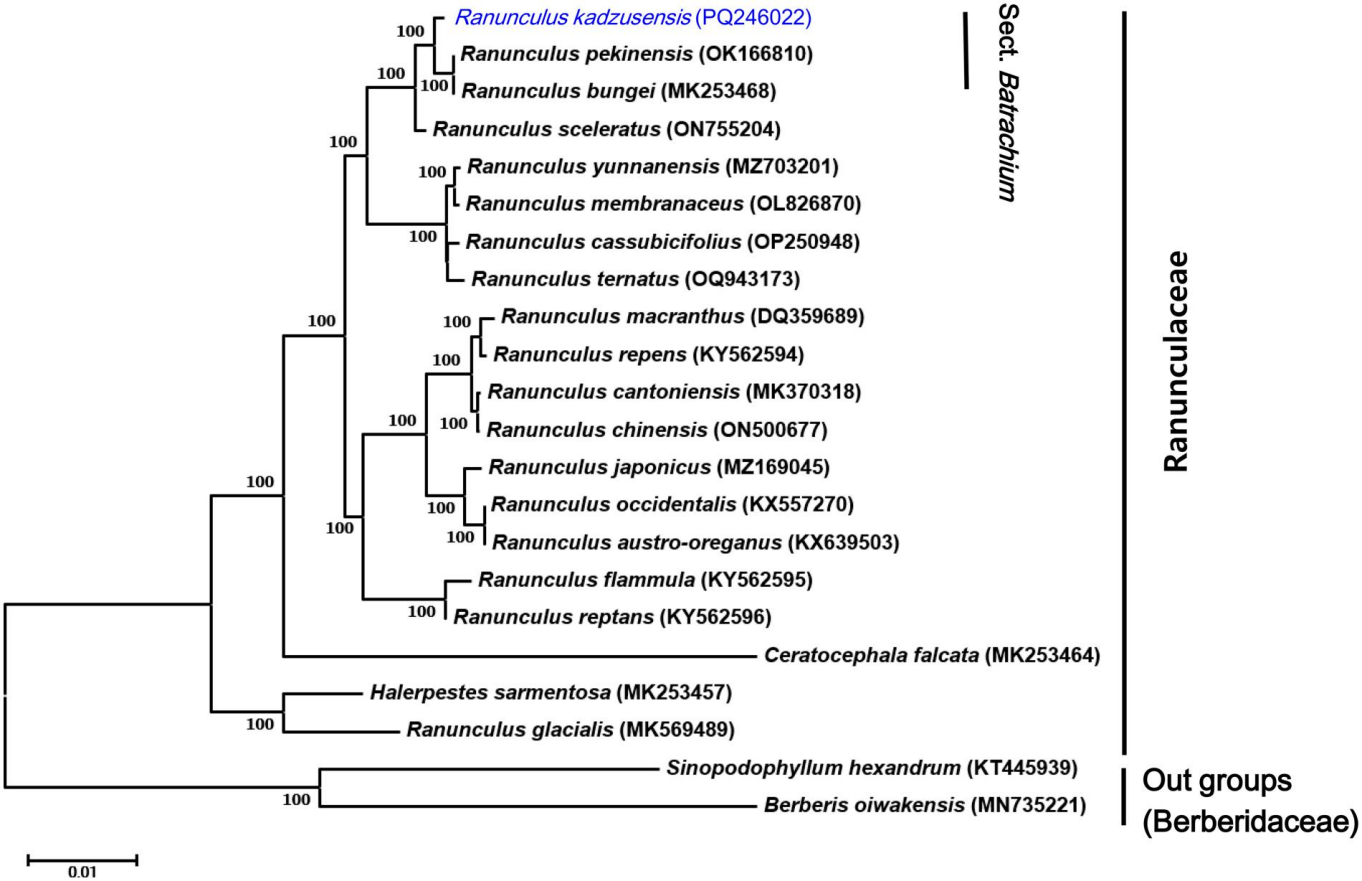
Phylogenetic tree based on the concatenated sequences of 67 protein-coding genes in 22 species by maximum-likelihood (ML). *Ranunculus kadzusensis* (PQ246022) was marked in bold. The following sequences were used: *Ranunculus pekinensis* OK166810 (Liu et al. 2022), *Ranunculus bungei* MK253468 (He et al. 2019), *Ranunculus sceleratus* ON755204 (Kim et al. 2023), *Ranunculus yunnanensis* MZ703201 (Rao et al. 2022), *Ranunculus membranaceus* OL826870 (Ren et al. 2023), *Ranunculus cassubicifolius* OP250948 (Karbstein et al. 2022), *Ranunculus ternatus* OQ943173 (Qiao et al. 2023), *Ranunculus macranthus* DQ359689 (Raubeson et al. 2007), *Ranunculus repens* KY562594 (Kim et al. 2023), *Ranunculus cantoniensis* MK370318 (Li et al. 2019), *Ranunculus chinensis* ON500677 (Li et al. 2019), *Ranunculus japonicus* MZ169045 (Zeng et al. 2021), *Ranunculus occidentalis* KX557270 (Kim et al. 2023), *Ranunculus austro-oreganus* KX639503 (Kim et al. 2023), *Ranunculus flammula* KY562595 (Qiao et al. 2023), *Ranunculus reptans* KY562596 (Kim et al. 2023), *Ranunculus glacialis* MK569489. *Ceratocephala falcata* MK253464, *Halerpestes sarmentosa* MK253457, as for an outgroup, *Sinopodophyllum hexandrum* KT445939 (Li and Guo 2015), *Berberis oiwakensis* MN735221 (Xiao et al. 2020) from Berberidaceae is included.

### Phylogenetic analysis

The phylogenetic analysis with 19 representative species in the suborder Ranunculaceae, including the newly completed *R. kadzusensis,* has illustrated that each representative species exhibits a monophyletic relationship with a bootstrap supporting value from 100% (Figure 3). Among these clades, the newly reported *R. kadzusensis* (158,301 bp) exhibited a closer relationship with *R. pekinesis* (156,139 bp) and *R. bungei* (156,082 bp). The chloroplast genome sequence of *R. kadzusensis* is a valuable resource for genome evolution and taxonomy research of the Ranunculaceae genus. A phylogenetic analysis was conducted to clarify the phylogenetic position of *R. kadzusensis* within the genus *Ranunculus*. The resulting phylogenetic tree demonstrated high bootstrap values at most nodes, supporting the robustness of the analysis and aligning well with previous studies. Within section *Batrachium*, *R. kadzusensis* was identified as closely related to *R. pekinensis* and *R. bungei.* It clearly shows the molecular phylogenetic position of *R. kadzusensis,* highlighting its relationship to other species within section *Batrachium* (Figure 3, Table S2).

## Discussion and conclusions

*Ranunculus* is a taxonomically challenging genus due to the presence of morphologically similar species, making species identification difficult. When combined with morphological analyses, chloroplast genome data enable more precise classification and differentiation. *R. kadzusensis* shares key characteristics with aquatic and semi-aquatic species in the section *Batrachium* (Wang and Tamura 2001). In this study, we confirmed that *R. kadzusensisis* closely related to *R. pekinensis* and *R. bungei* (Wang et al. 2010) in the section *Batrachium* while being distinct from *R. sceleratus* in section Hecatonia (Uni. of North Carolina, FSUS Database. 2024). These findings prove that *R. kadzusensis* is an independent species within the section *Batrachium*. This result serves as an essential reference for accurate species identification and classification. In particular, our study establishes a key criterion for distinguishing *R. kadzusensis* from *R. pekinensis* and *R. bungei*, helping to prevent misidentification among these species.

## Supplementary Material

Supplementary Figure.docx

## Data Availability

The data from this study are openly accessible in GenBank of NCBI at https://www.ncbi.nim.nih.gov/ under the accession number PQ246022. The associated BioProject, SRA, and BioSample numbers are PRJNA1152639, SRR30407466, and SAMN43372901 respectively.
