# Supplementary material for: The complete chloroplast genome sequence and phylogenetic analysis of Ranunculus kadzusensis Makino 1929, an endangered species in Korea
Source: Mitochondrial DNA B Resour. 2025 Jun 17;10(7):568–72. doi: 10.1080/23802359.2025.2519219 (PMC12175185; doi:10.1080/23802359.2025.2519219)

Supplementary Figure

**Figure S1. The coverage depth of the chloroplast genome of *Ranunculus kadzusensis.***


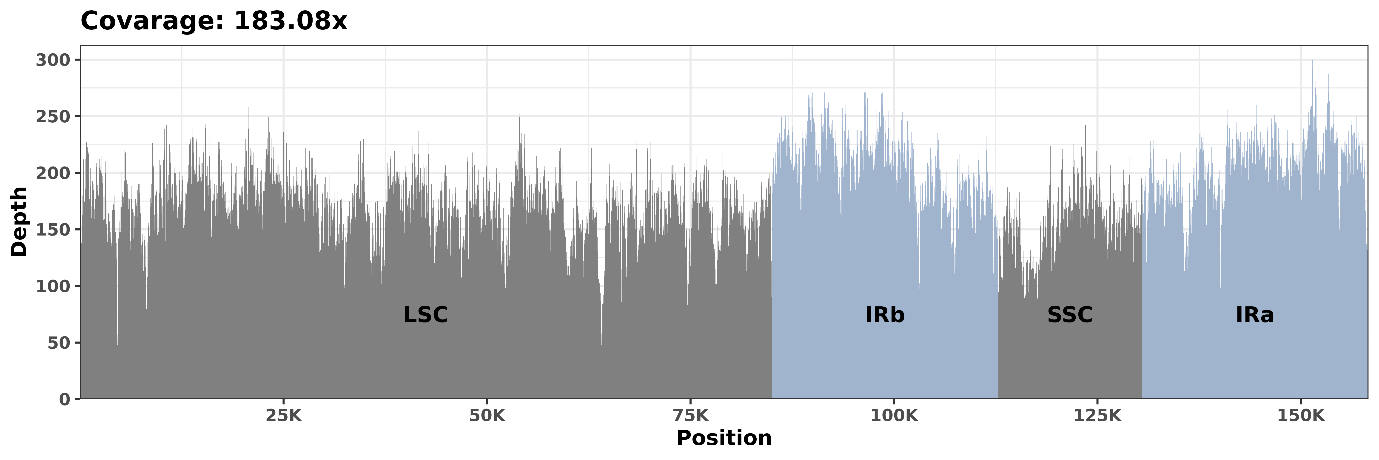


**Figure S2. Schematic maps of cis-splicing genes in the chloroplast genome of *Ranunculus kadzusensis* generated using PMGmap View.**


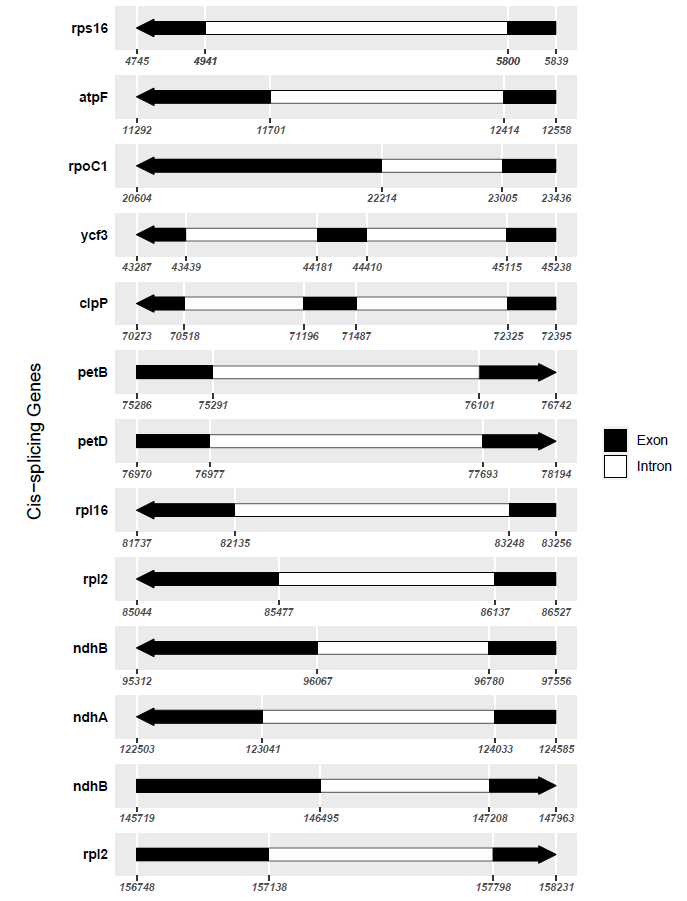


**Figure S3. Schematic maps of the trans-spliced gene *rps12*.**


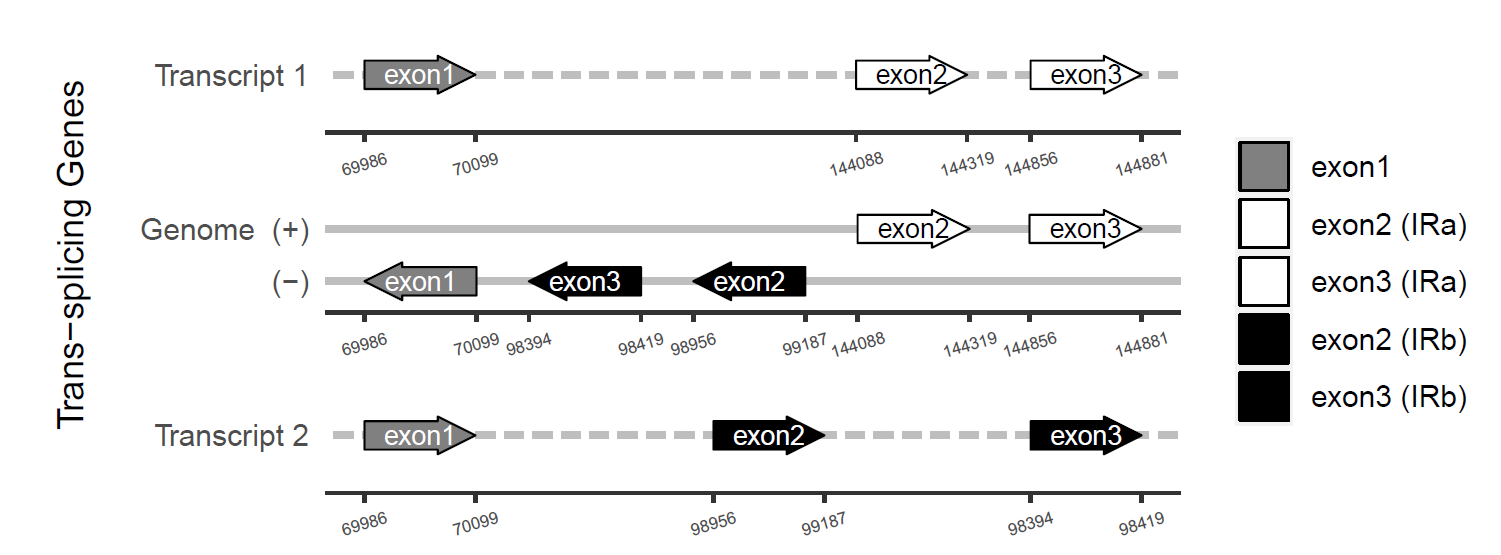

Supplement: Supplementary Figure.docx [file TMDN_A_2519219_SM5734.docx]
